# Splenic Artery Pseudoaneurysm With Fistulization to the Transverse Colon: A Case Report

**DOI:** 10.7759/cureus.114856

**Published:** 2026-08-20

**Authors:** Meagan Mitchell, Christina A Schott, Gustavo Rodriguez, Marian Khalili, Matthew Ng

**Affiliations:** 1 Department of Surgery, George Washington University School of Medicine and Health Sciences, Washington, USA; 2 Department of Surgery, George Washington University Hospital, Washington, USA

**Keywords:** endovascular embolization, fistulization, gastrointestinal bleeding, hemorrhagic shock, splenic artery pseudoaneurysm

## Abstract

Splenic artery pseudoaneurysms (SAPs) are rare occurrences, most commonly secondary to pancreatitis, abdominal trauma, or postoperative/iatrogenic causes. The most common presenting symptom is abdominal pain, but it may also be hematochezia, melena, or hematemesis. SAP rupture or fistulation causes massive hemorrhage leading to hemorrhagic shock and possible death if left untreated. The timely diagnosis and treatment of SAPs are therefore crucial in managing these patients. Here, we present a case of a female in her 80s presenting with rectal bleeding, diagnosed with SAP with fistulization to the transverse colon as seen on contrasted abdominopelvic CT. She was treated with endovascular embolization, which successfully resolved the bleeding, and subsequent splenectomy and splenic flexure colectomy. The patient’s postoperative course was complicated by urinary retention due to presumed UTI, left-sided pleural effusion, mitral regurgitation, and tricuspid regurgitation. Once the complications resolved, the patient did well with symptom resolution. Early diagnosis is crucial for the management of these patients. Due to the nonspecific presentation, a high level of suspicion should prompt further evaluation to prevent adverse outcomes. Fistulization of SAPs to surrounding structures could lead to complications, as was seen with this patient. However, endovascular embolization of the SAP with subsequent segmental resection of the involved colon proved to be a successful treatment.

## Introduction

Splenic artery pseudoaneurysms (SAPs) are a rare entity estimated to occur in less than 1% of the general population, with less than 200 cases reported in the literature. Pancreatitis, abdominal trauma, and postoperative/iatrogenic causes can also increase the risk of SAP [1-4]. The most common presenting symptom is abdominal pain, but patients may also present with hematochezia, melena, or hematemesis [2]. If left untreated, SAPs have up to a 76.3% risk of rupture and a 90% risk of mortality, with most of them being diagnosed postmortem. This is much higher than true aneurysms due to SAPs having a false vessel wall, which disrupts the artery wall's normal structure. The absence of a complete arterial wall around the blood flow means that the pseudoaneurysm can more easily burst, especially if it is under high pressure or if there are additional contributing factors like trauma or infection [5,6]. Additionally, pseudoaneurysms can grow rapidly, and diameter is not a reliable predictor of rupture risk [5,6]. The timely diagnosis and treatment of SAPs is therefore crucial in the management of these patients. We present a case of SAP with fistulization to the transverse colon in a patient presenting with rectal bleeding, treated with embolization and subsequent splenectomy and splenic flexure colectomy. This article was previously presented as a meeting abstract at the 2024 American Society of Colon and Rectal Surgeons (ASCRS) Annual Scientific Meeting on June 1, 2024.

## Case presentation

A female in her 80s presented to the emergency department of a level-II trauma center after three episodes of bright red blood per rectum. She reported associated vague abdominal pain, nausea, intermittent vomiting, two to three near syncopal episodes, lightheadedness, and fatigue over the past two weeks. She denied hematemesis or melena at that time. Her medical conditions included atrial fibrillation rate controlled on carvedilol and digoxin with Eliquis® on hold, hyperlipidemia, and hypertension. Hemoglobin (Hgb) on admission was 6.5 g/dL and she was hypotensive. CT of the abdomen and pelvis revealed a splenic artery pseudoaneurysm adjacent to the splenic flexure of the colon. The patient underwent embolization by interventional radiology (IR) six days after presentation, with successful resolution of bleeding. The patient required seven units of packed red blood cells (PRBC) to maintain Hgb above 7. Further evaluation with colonoscopy on the same day showed a splenic artery aneurysm with retroperitoneal phlegmon fistulization of the colon and no ongoing bleeding. Her abdominal pain resolved, she tolerated a regular diet, and was having non-bloody bowel movements. The decision was made by her care team and family to transfer to a tertiary center for evaluation by hepato-pancreato-biliary (HPB) surgery. On admission to our center, the patient was doing well symptomatically with no abdominal pain, nausea, vomiting, or lightheadedness. Laboratory test results drawn on admission are shown in Table 1.

**Table 1:** Laboratory test results upon admission to tertiary care center after embolization Reference ranges reported from Harrison's Principles of Internal Medicine [7].

Lab test	Lab value	Reference range
White blood cell (WBC) count	13.05 x10^3^/mcl (high)	4,500 to 11,000 cells per microliter (mcl)
Red blood cell (RBC) count	3.05 x10^3^/mcl (low)	4.2-5.4 x10^3^ cells per microliter (mcl)
Hemoglobin (Hgb)	9.4 g/dL (low)	12–16 grams per deciliter (g/dL)
Hematocrit (Hct)	28.6% (low)	37–47%
Prothrombin time (PT)	13.5 seconds	11-13.5 seconds
International normalized ratio (INR)	1.06	1–1.4
Partial thromboplastin time (PTT)	39.9 seconds (high)	25–37 seconds

**Figure 1:**
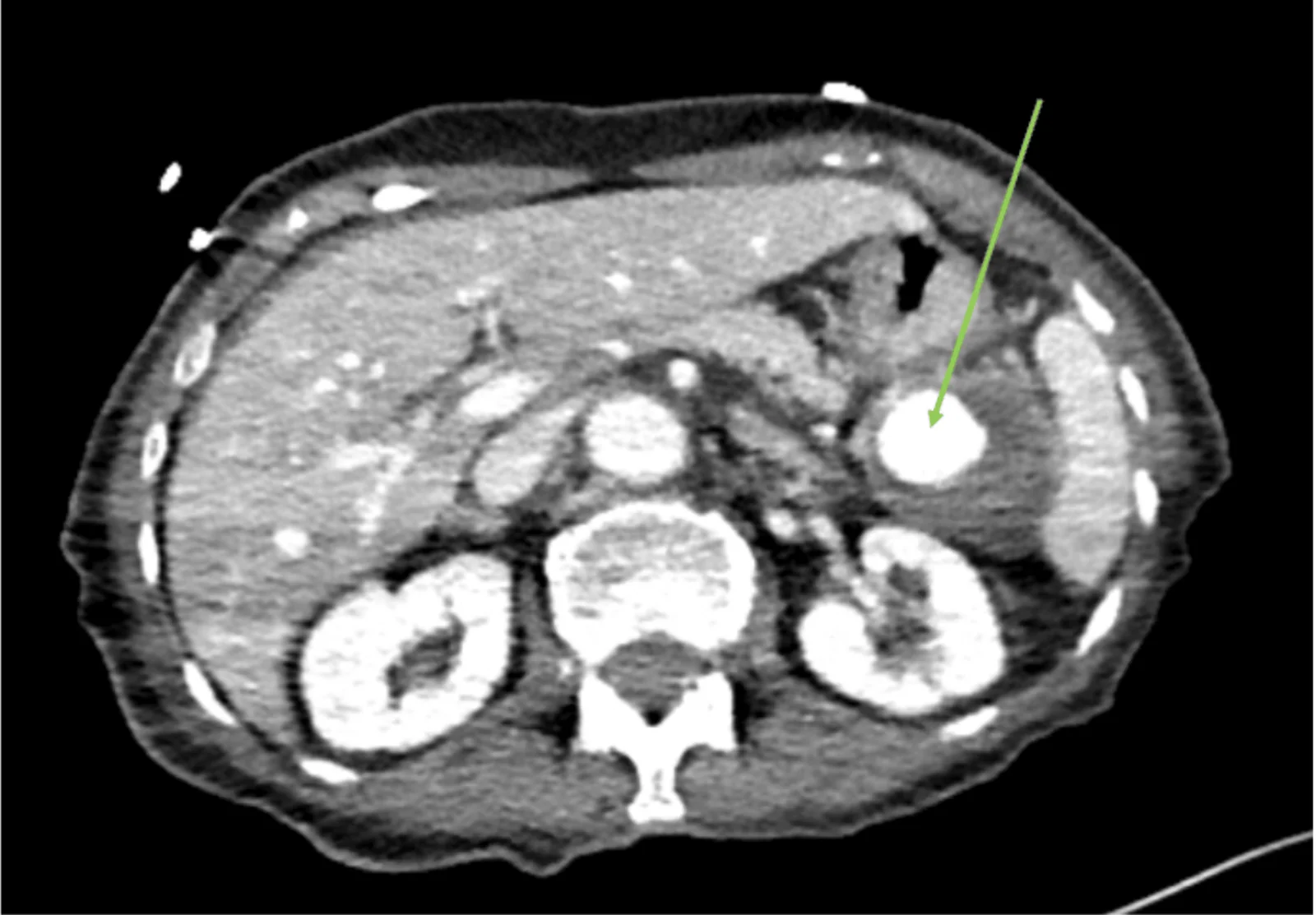
Axial arterial-phase contrast-enhanced CT scan of the abdomen The CT image is showing the SAP (arrow) with diffuse contrast opacification of the transverse colon, raising concern for a fistulous connection resulting in hemorrhage into the colonic lumen.

A repeat CT angiogram showed embolization coils in the area of the distal splenic artery surrounded by an 8 cm fluid collection with apparent communication between this collection and the splenic flexure of the colon. No active extravasation of contrast from the vicinity of the previously embolized splenic artery was seen (Figure 1).

**Figure 2:**
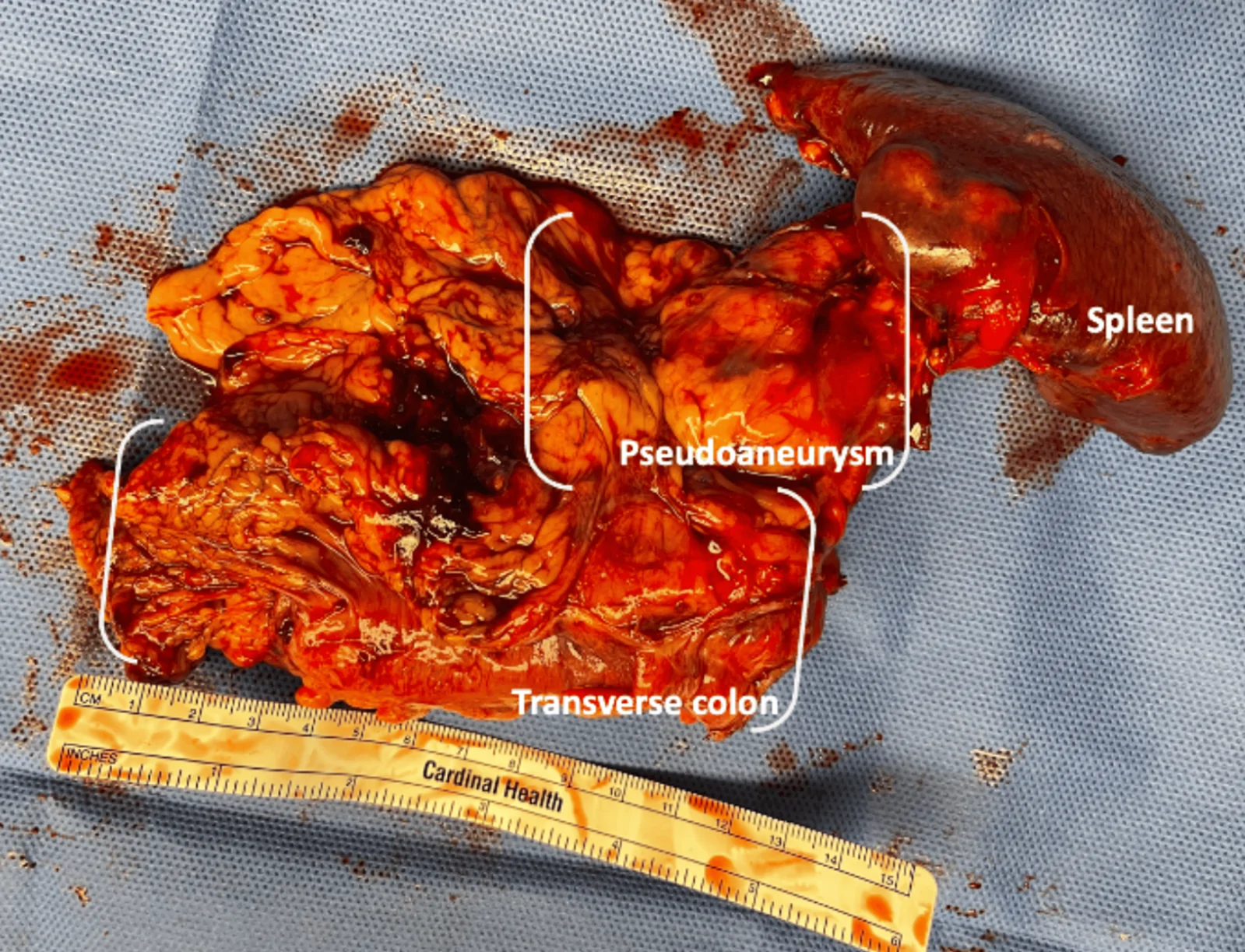
Image of the resected distal transverse colon Image of the resected distal transverse colon, splenic flexure, and spleen revealing a pseudoaneurysm.

She underwent a laparoscopic-assisted splenectomy and splenic flexure colectomy with HPB and colorectal surgery teams. The splenic flexure and spleen were mobilized before resecting proximal to the splenic artery and vein where the aneurysm was. The left white line of Toldt was dissected with electrocautery and the mesocolon was mobilized medially. The descending colon was fully mobilized for evisceration through the hand port to allow for anastomosis. The fistula was visualized and palpated before proceeding with distal transection in an area of healthy, viable descending colon and proximal transection at the mid-transverse colon (Figure 2). Both ends of the colon were reapproximated, and a side-to-side, functional end-to-end anastomosis was performed. The anastomosis was then replaced into the abdomen, and hemostasis was ensured. A Jackson-Pratt (JP) surgical drain was placed at the left lower quadrant port site. There were no immediate complications.

The patient’s postoperative course was complicated by a urinary tract infection (UTI), pleural effusions, a peripancreatic abscess, and newly identified mitral and tricuspid regurgitation. Urinary retention secondary to UTI resolved following treatment with ceftriaxone. On postoperative day 7, the patient developed worsening leukocytosis, with the white blood cell count increasing from 18 × 10⁹/L to 23 × 10⁹/L. Computed tomography demonstrated a left pleural effusion and a 3.2-cm peripancreatic fluid collection concerning for abscess formation. The patient subsequently underwent ultrasound-guided thoracentesis and interventional radiology-guided drainage of the peripancreatic collection with drain placement. Following source control and initiation of targeted antibiotic therapy, the white blood cell count decreased from a peak of 33 × 10⁹/L to 15 × 10⁹/L. Cardiac evaluation revealed mitral and tricuspid regurgitation with preserved left ventricular ejection fraction. The patient’s respiratory status improved with diuresis and drainage of the pleural effusion. After resolution of these complications, she was discharged home in stable condition with complete resolution of her presenting symptoms.

## Discussion

The risk of developing SAPs increases with a history of acute or chronic pancreatitis, pancreatic pseudocyst, abdominal trauma, and infection [1,2]. Release of pancreatic enzymes or other inflammatory processes causes elastin breakdown and weakens the vessel wall, allowing for hematoma formation between the tunica media and tunica adventitia [5]. Although this patient had primary risk factors for splenic artery aneurysms (SAAs), namely hypertension, hyperlipidemia, and being female, she had no history of pancreatic disease or reported history of trauma that would have increased her risk for SAP development. Although the cause of her SAP was unclear, she presented with fistulization of the SAP to the transverse colon, causing hematochezia, a known complication of SAPs. Fistulization to other structures, such as the stomach, pancreatic duct, or duodenum, has also been described [3]. Fistulization of the SAP to the colon could lead to massive hemorrhage, leading to hemorrhagic shock and possible death if left untreated.

To prevent this potentially fatal complication of SAPs, early detection and diagnosis are crucial. The tools most commonly used for diagnosing SAAs are abdominal ultrasound, abdominal and pelvic CT, magnetic resonance imaging, and magnetic resonance angiography. While the gold standard for diagnosis is digital subtraction angiography, the recommended initial diagnostic tool is computed tomography angiography [5,8]. Radiologists identifying these vascular pathologies must recognize the clinical significance. In this case, timely diagnosis via abdominopelvic CT was imperative in preventing rupture and providing timely treatment. A collaborative care plan must be devised by interventional radiology (IR) or vascular surgery, HPB surgery, and colorectal surgery teams to initially control the hemorrhage and definitively treat the affected organs.

Multiple treatment strategies have been used in the treatment of SAPs. In the Society for Vascular Surgery Clinical Practice Guidelines, treatment of nonruptured splenic artery pseudoaneurysms of any size in patients of acceptable risk is strongly recommended based on a moderate level of evidence because of the high possibility of rupture [5]. Surgical treatment with aneurysmectomy, including splenectomy with or without partial pancreatectomy, has been the treatment of choice for SAPs. Endovascular techniques involving transcatheter embolization with coils or other materials are becoming the preferred treatment due to lower risk and spleen preservation [2,9,10]. Treatment with percutaneous thrombin injection has also been described [1,5,8]. In patients with associated pseudocyst, which may be as high as 41%, surgical excision is a more reliable treatment than embolization [3]. In our patient, initial embolization with coils allowed for control of GI hemorrhage, and subsequent splenectomy and splenic flexure colectomy resected the fistulization to the transverse colon and removed the infarcted spleen.

## Conclusions

We present a case of a patient with SAP fistulizing to the transverse colon. Given that this is a rare but potentially life-threatening condition, early diagnosis is crucial for the management of these patients. As the typical presenting symptoms of SAP are shared with multiple other pathologies, a high level of suspicion should prompt further evaluation to prevent adverse outcomes. Fistulization of SAPs to surrounding structures could lead to complications as was seen with this patient. However, endovascular embolization of the SAP with subsequent segmental resection of the involved colon proved to be a successful treatment of this patient’s SAP. 
